# Prevalence of left ventricular diastolic dysfunction by cardiac magnetic resonance imaging in thalassemia major patients with normal left ventricular systolic function

**DOI:** 10.1186/s12872-019-1235-8

**Published:** 2019-11-06

**Authors:** Benjaporn Chinprateep, Nithima Ratanasit, Yodying Kaolawanich, Khemajira Karaketklang, Pairash Saiviroonporn, Vip Viprakasit, Rungroj Krittayaphong

**Affiliations:** 1 Division of Cardiology, Department of Medicine, Faculty of Medicine, Siriraj Hospital, Mahidol University, 2 Wanglang Road, Bangkoknoi, Bangkok, 10700 Thailand; 2 Department of Medicine, Faculty of Medicine, Siriraj Hospital, Mahidol University, Bangkok, Thailand; 3 Department of Radiology, Faculty of Medicine, Siriraj Hospital, Mahidol University, Bangkok, Thailand; 4 Division of Hematology and Oncology, Department of Pediatrics, Faculty of Medicine, Siriraj Hospital, Mahidol University, Bangkok, Thailand

**Keywords:** Thalassemia, Left ventricular diastolic dysfunction, Left ventricular diastolic dysfunction, Cardiac magnetic resonance imaging

## Abstract

**Background:**

The leading cause of mortality of thalassemia major patients is iron overload cardiomyopathy. Early diagnosis with searching for left ventricular diastolic dysfunction before the systolic dysfunction ensued might yield better prognosis. This study aimed to define the prevalence of the left ventricular diastolic dysfunction (LVDD) in thalassemia major patients with normal left ventricular systolic function and the associated factors.

**Methods:**

Adult thalassemia major patients with normal left ventricular systolic function who were referred for cardiac T2* at Siriraj Hospital – Thailand’s largest national tertiary referral center – during the October 2014 to January 2017 study period. Left ventricular diastolic function was defined by mitral valve filling parameters and left atrial volume index using CMR. Patients with moderate to severe valvular heart disease, pericardial disease, or incomplete data were excluded. Baseline characteristics, comorbid diseases, current medication, and laboratory results were recorded and analyzed.

**Results:**

One hundred and sixteen patients were included, with a mean age of 27.5 ± 13.5 years, 57.8% were female, and 87.9% were transfusion dependent. Proportions of homozygous beta-thalassemia and beta-thalassemia hemoglobin E were 12.1 and 87.9%, respectively. The baseline hematocrit was 26.3 ± 3.3%. The prevalence of LVDD was 20.7% (95% CI: 13.7–29.2%). Cardiac T2* was abnormal in 7.8% (95% CI: 3.6–14.2%). Multivariate analysis revealed age, body surface area, homozygous beta-thalassemia, splenectomy, heart rate, and diastolic blood pressure to be significantly associated with LVDD.

**Conclusions:**

LVDD already exists from the early stages of the disease before the abnormal heart T2 * is detected. Homozygous beta-thalassemia and splenectomy were strong predictors of LVDD. These data may increase awareness of the disease, especially in the high risk groups.

## Introduction

The leading cause of mortality of thalassemia major patients is iron overload cardiomyopathy. Since current diagnosis is made by evaluation of T2* by cardiac magnetic resonance (CMR) imaging [1], the myocardium is already involved by the iron. Some patients are detected in late of the disease with impaired left ventricular systolic function, and unfavorable outcomes. Previous studies in iron overload cardiomyopathy found left ventricular diastolic dysfunction could be detected before the systolic dysfunction ensued [2]. And in other diseases, such as coronary artery disease and heart failure, diastolic dysfunction was reported to be associated with increased morbidity and mortality [3–5]. Thus, left ventricular diastolic function is important for early detection of the disease and treatment, along with better prognosis.

Previous studies of left ventricular diastolic function in thalassemia major patients have various in results in prevalence and associated factors [6–10]. The reported prevalence of LVDD ranged widely from 7.9 to 100%, and most cases demonstrated restrictive filling pattern [8, 9, 11]. The prevalence of iron overload cardiomyopathy also varied depending on the standard of care that the patients received in that region and the era of the report. The prevalence could be more than 50% in some previous reports [9, 12]. In the tertiary-care setting with well-controlled iron chelation therapy in the current era and available CMR system for the detection and assessment of cardiac involvement, the prevalence of cardiac iron overload may be less than 10% [13]. Left ventricular diastolic dysfunction usually precede systolic dysfunction and heart failure [2]. Left ventricular diastolic dysfunction cannot be completely explained by cardiac iron overload since previous data indicated that parameters for mitral valve filling were not well correlated with cardiac T2* [10] and chelation treatment did not completely protect patients from left ventricular diastolic and systolic dysfunction [8]. Previous study showed that left ventricular diastolic function can be improved by medication in patients with thalassemia [14]. Most of the studies used echocardiogram for evaluation of the left ventricular diastolic function. Biomarker such as brain natriuretic peptide can detect early cardiac involvement in thalassemia patients but it may not be cost effective to use as a screening [15]. While current diagnosis of cardiac involvement by the iron overload is done by CMR and many patients were requested to undergo CMR without echocardiogram. Parameters for assessment of diastolic function by CMR has been shown to be well correlated with echocardiographic parameters [16]. This study aimed to define the prevalence of left ventricular diastolic dysfunction (LVDD) in thalassemia major patients with normal left ventricular systolic function. The associated factors of LVDD was also analyzed. In this study, CMR parameters were used for the assessment of left ventricular diastolic function.

## Patients and methods

### Study population

The study protocol was approved by the Siriraj Institutional Review Board of the Faculty of Medicine Siriraj Hospital, Mahidol University, Bangkok Thailand, and all patients provided written informed consent to participate. Adult thalassemia major patients (age ≥ 18 years) with normal LV systolic function (LV ejection fraction ≥55% by CMR [17]) who were evaluated for cardiac T2* at Siriraj Hospital during 1 October 2014 to 4 January 2017 were included. Patients with moderate to severe valvular heart disease, pericardial disease, contraindication for magnetic resonance imaging (MRI) or claustrophobia, or incomplete data were excluded. Patient data was retrieved from electronic medical records and from the CMR database. Baseline characteristics, comorbid diseases, current medication, and laboratory results were recorded and analyzed.

### Cardiac magnetic resonance (CMR) imaging technique

**Fig. 1:**
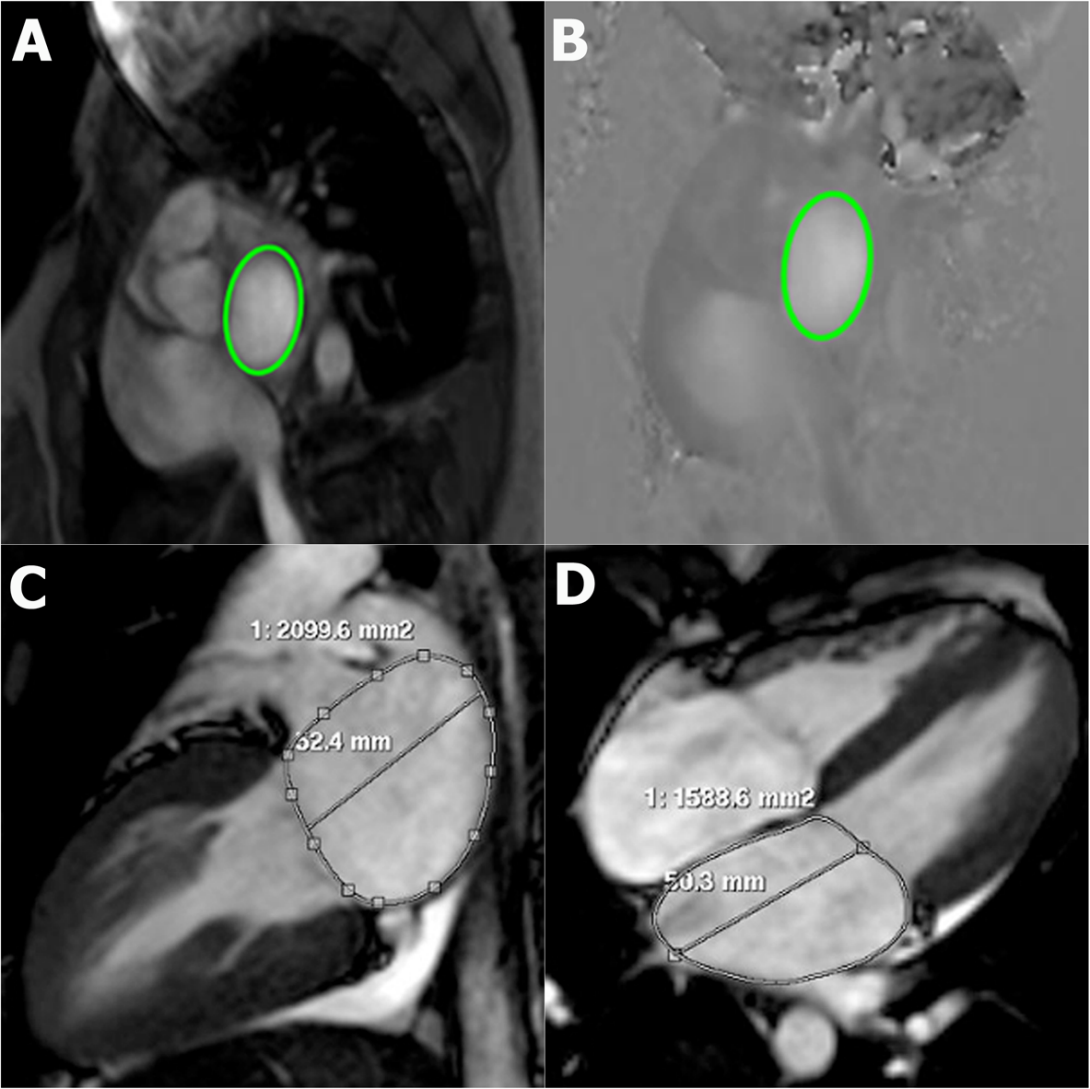
CMR technique – two-dimensional phase contrast imaging at mitral annular plane. Region of interest was drawn from magnitude image (**a**), flow and peak velocity were calculated from phase image (**b**). Mitral velocity-time curve yielded E, A, and DT, which represented the time from peak of E wave to the point intersected by the downslope line drawn from peak of E wave. Left atrial (LA) volume was calculated by area-length method in two-chamber (**c**) and four-chamber (**d**) views. The LA area was contoured at the end systole, but the left atrial appendage and pulmonary veins were excluded. LA length was drawn from midpoint of the mitral annulus to the opposite LA wall

Cardiac function and T2* were performed on a 1.5 T Achieva-XR Quasar-Dual-Gradient System (Philips Medical Systems, Best, The Netherlands) with a 5-element cardiac coil. Steady-state free precession, breath-hold technique was performed for cardiac function evaluation. For cardiac function, cines of 4-chamber, 2-chamber, and short-axis views from apex to atrioventricular valve ring were acquired. The CMR parameters were, as follows: 8 mm slice thickness for 10–12 slices without gap in between, repetition time (TR) 3.4 ms, echo time (TE) 1.7 ms, and flip angle 60°. LV ejection fraction (LVEF), LV end-diastolic volume (LVEDV), LV systolic volume (LVSV), and LV mass were calculated as previously described by Krittayaphong, et al. [17]. Left atrial (LA) volume was calculated by area-length method [18]. Two-chamber and four-chamber views were used. The LA area was contoured at the end systole, but the left atrial appendage and pulmonary veins were excluded. LA length was drawn from the midpoint of the mitral annulus to the opposite LA wall (Fig. 1). LA volume was calculated using the following formula:

LA volume = 0.85 x LA area (2-chamber view) x LA area (4-chamber view)/LA length.

(The shortest LA length from the 2-chamber view and 4-chamber view was used in the formula)

Cardiac T2* was performed with gradient-echo, black blood technique. Single short-axis mid-ventricular slice with 8 TE from 2.60–16.74 ms with each step of 2.02 ms was used. CMR parameters for T2* were, as follows: slice thickness 10 mm, TR 18 ms, flip angle 20°, field of view (FOV) 400 × 400 mm, and voxel size 3.0 × 1.5 × 10 mm^3^. The 8 images were quantitatively analyzed by pixel-wise method for all segments, and then the septum was chosen. The median T2* value was reported as abnormal if the T2* was ≤20 ms. This method was well-validated and had good reproducibility [19–23]. Diastolic function was analyzed from velocity-time curve and volume-time curve at mitral valve inflow on the mitral annular plane, which was derived from the cross-sectional line of mitral annulus in 4-chamber and 2-chamber view at end-diastolic phase. Two-dimensional phase contrast imaging at the mitral annular plane with retrospective ECG gating was analyzed. The parameters were TR 6.1 ms, TE 3.7 ms, slice thickness 8 mm without gap between slices, flip angle 12°, FOV 320 × 320 mm, included 40 phases per a cardiac cycle, and velocity encoding 100 cm/s. Region of interest was drawn from magnitude image, and flow and peak velocity were calculated from phase image (Fig. 1). The mitral volume-time curve yielded early peak filling rate (EPFR) and late peak filling rate (LPFR), and the velocity-time curve yielded early diastolic filling velocity (E), late diastolic filling velocity (A), and deceleration time (DT), which is the time from peak of E wave to baseline. The method was adapted from previous study, which showed good correlation with echocardiographic parameters [24–27].

**Fig. 2:**
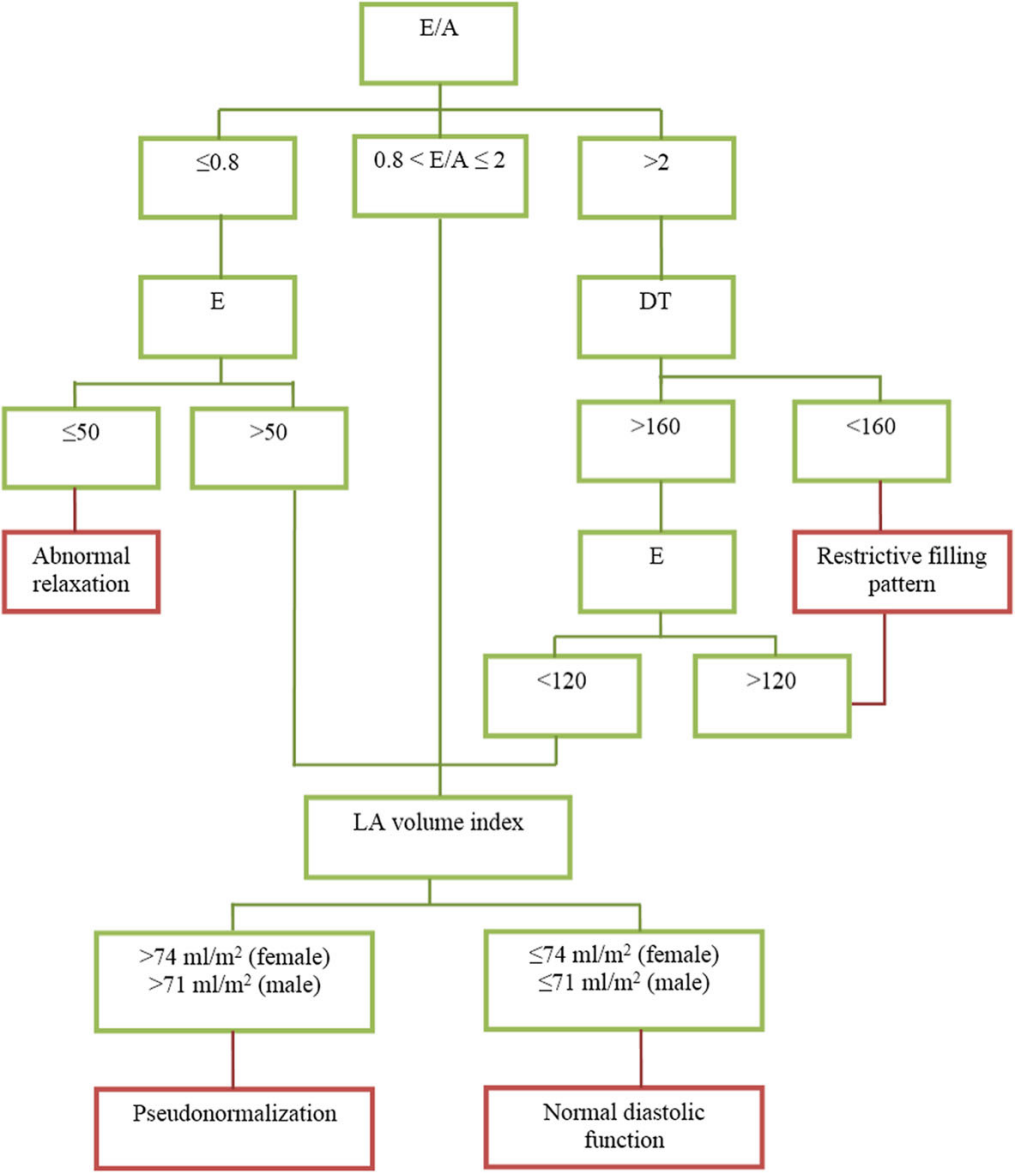
Left ventricular diastolic function was defined as abnormal relaxation or restrictive filling pattern by the condition described. If it could not be determined, left atrial volume index was used to define pseudonormalization or normal diastolic function. E velocity = early diastolic filling velocity, E/A = E/A ratio, DT = deceleration time, LA = left atrial

We use CMR, not echocardiography, to determine LVDD in the present study. The criteria for LVDD was modified from the ASE 2009 criteria [28], which focused on parameters on mitral valve filling, and LAVI as suggested by the ASE 2016 criteria [29]. LV diastolic function was defined as abnormal relaxation if E/A ratio ≤ 0.8 and E ≤ 50 cm/s, or as restrictive filling pattern if E/A ratio > 2 and DT < 160 ms or DT > 160 ms with E > 120 cm/s. Left atrial volume index (LAVI) was used to further classify diastolic function in selected patients as shown in Fig. 2 [30]. If the LAVI was abnormally high (> 74 ml/m^2^ in female, and > 71 ml/m^2^ in male) [18], pseudonormalization of LV diastolic function was diagnosed.

### Statistical analysis

Baseline characteristics were summarized using descriptive statistics. Frequency and percentage were used to describe categorical variables. Continuous variables were reported as mean ± standard deviation for normally distributed variables, and as median and interquartile range (25th and 75th percentile) for non-normally distributed variables. Kolmogorov-Smirnov test was used to evaluate for normal distribution. The prevalence of LVDD is described as percentage and 95% confidence interval. Association between continuous variables and LVDD was determined using Student’s *t*-test or Mann-Whitney U test. For categorical variables, chi-square test or Fisher’s exact test was used to test for association. Variables associated with LVDD at a *p*-value less than 0.2 were entered into univariate analysis. Variables found to be significantly associated with LVDD in univariate analysis were entered into multivariate analysis using forward stepwise multiple logistic regression.

Intraclass correlation coefficient (ICC) was performed in 10 patients to measure intraobserver and interobserver reliability. For all tests performed, a two-tailed *p*-value < 0.05 was considered to denote statistical significance. The statistical software package SPSS (version 18; SPSS, Inc., Chicago, IL, USA) was employed for all data analyses.

## Results

There were 123 patients eligible for inclusion during the 1 October 2014 to 4 January 2017 study period. However, diastolic function could not be interpreted in 7 patients due to absence of A or fusion of E and A waves, so those patients were excluded from the study. The remaining 116 patients were enrolled. Baseline characteristics of study population were shown in Table 1. Cardiac magnetic resonance imaging parameters of study population were compared between normal diastolic function group and diastolic dysfunction group as in Table 2.

**Table 1:** Baseline characteristics of study population

Variables	Total (*n* = 116)	Normal diastolic function (*n* = 92)	Diastolic dysfunction (*n* = 24)	*p*-value
Age (years)	27.46 ± 13.52	26.13 ± 10.17	32.54 ± 21.70	0.171
Female gender	67 (57.76%)	54 (58.70%)	13 (54.17%)	0.689
Body weight (kg)	47.46 ± 10.02	48.28 ± 10.38	44.29 ± 7.91	0.082
Body surface area (m^2^)	1.43 ± 0.19	1.44 ± 0.20	1.37 ± 0.16	0.101
SBP (mmHg)	109.62 ± 14.88	109.97 ± 12.51	108.29 ± 22.04	0.723
DBP (mmHg)	61.56 ± 11.50	62.89 ± 11.23	56.46 ± 11.32	***0.014***
Heart rate (bpm)	83.11 ± 11.72	84.80 ± 11.85	76.63 ± 8.65	***0.002***
Homozygous beta thalassemia	14 (12.07%)	7 (7.61%)	7 (29.17%)	***0.009***
Hemoglobin E/beta thalassemia	102 (87.93%)	85 (92.39%)	17 (70.83%)	***0.009***
Splenectomy (n = 116)	28 (24.13%)	18 (19.56%)	10 (41.67%)	***0.026***
Transfusion dependent	95 (81.90%)	77 (83.70%)	18 (75.00%)	0.374
Total transfusion in 1 year (ml); median (IQR)	175.41 ± 61.13 4 (4–4)	177.74 ± 64.40 4 (4–4)	176.63 ± 45.90 4 (3–4)	0.944
Diabetes mellitus	11 (9.48%)	8 (8.70%)	3 (12.50%)	0.695
Hypothyroidism	11 (9.48%)	7 (7.61%)	4 (16.67%)	0.235
Hypertension	1 (0.86%)	0 (0.00%)	1 (4.17%)	0.207
Medication use				
- Deferiprone	57 (49.14%)	46 (50.00%)	11 (45.83%)	0.716
- Deferasirox	43 (37.07%)	33 (35.87%)	10 (41.67%)	0.601
- Deferoxamine	39 (33.62%)	34 (36.97%)	5 (20.83%)	0.136
- Beta blockers	7 (6.03%)	4 (4.35%)	3 (12.50%)	0.154
- ACEI/ARB	2 (1.72%)	1 (1.09%)	1 (4.17%)	0.372
- Statin	2 (1.72%)	1 (1.09%)	1 (4.17%)	0.372
- Diuretics	2 (1.72%)	1 (1.09%)	1 (4.17%)	0.372
- Aspirin	14 (12.07%)	10 (10.87%)	4 (16.67%)	0.484
Hemoglobin (g/dl)	8.48 ± 1.21	8.53 ± 1.20	8.28 ± 1.27	0.361
Hematocrit	26.33 ± 3.26	26.44 ± 3.22	25.89 ± 3.44	0.467
Creatinine (mg/dl)	0.64 ± 0.17	0.64 ± 0.17	0.66 ± 0.17	0.462
Ferritin (mcg/l)	1440.0 (678.0–2575.50)	1411.00 (596.00–2411.00)	1502.50 (824.50–2694.00)	0.424
Liver iron concentration (mg Fe/g dry weight)	7.40 (3.65–15.45)	7.15 (2.95–14.55)	8.30 (4.90–23.75)	0.116

A *p*-value< 0.05 indicates statistical significance (bold and italic)

Data presented as mean ± standard deviation, median and interquartile range (IQR, 25th–75th percentile), or number and percentage

Abbreviations: *SBP* Systolic blood pressure, *DBP* Diastolic blood pressure, *bpm* Beats per minute, *ACEI/ARB* Angiotensin converting enzyme inhibitor/angiotensin receptor blocker, *Fe* Iron

**Table 2:** Cardiac magnetic resonance imaging parameters of study population

Variables	Total (n = 116)	Normal diastolic function (n = 92)	Diastolic dysfunction (n = 24)	*p*-value
LVEF (%)	63.59 ± 4.67	63.66 ± 4.47	63.33 ± 5.47	0.765
LVEDV (ml)	135.63 ± 34.15	132.17 ± 30.79	148.45 ± 43.12	0.093
LVESV (ml)	49.43 ± 14.20	47.88 ± 12.09	55.39 ± 19.81	0.087
LVMI (g/m^2^)	50.87 ± 13.40	49.52 ± 12.72	56.02 ± 14.91	***0.034***
LA area 2ch (mm^2^)	18.38 ± 4.61	17.64 ± 3.73	21.21 ± 6.38	***0.014***
LA length 2ch (mm)	45.01 ± 6.11	44.33 ± 5.42	47.63 ± 7.83	***0.018***
LA area 4ch (mm^2^)	20.59 ± 4.74	19.73 ± 3.84	23.92 ± 6.30	***0.004***
LA length 4ch (mm)	54.17 ± 6.79	53.11 ± 5.88	58.25 ± 8.48	***0.009***
LAVI (ml/m^2^)	50.37 ± 16.27	46.27 ± 10.48	66.06 ± 23.76	***0.001***
EPFR (ml/s)	383.26 ± 94.41	375.97 ± 82.55	411.19 ± 128.75	0.213
LPFR (ml/s)	210.30 ± 62.23	211.34 ± 59.56	206.31 ± 72.85	0.726
E velocity (cm/s)	61.76 ± 11.74	61.38 ± 10.86	63.23 ± 14.83	0.493
A velocity (cm/s)	42.21 ± 13.43	43.17 ± 13.96	38.54 ± 10.60	0.133
E/A	1.55 ± 0.42	1.49 ± 0.33	1.78 ± 0.60	***0.035***
Deceleration time (ms)	147.34 ± 32.95	147.94 ± 30.55	145.02 ± 41.58	0.701
Cardiac T2* (ms)	36.46 ± 8.96	36.65 ± 8.53	35.71 ± 10.61	0.649
Abnormal cardiac T2* (< 20 ms)	9 (7.76%)	7 (7.61%)	2 (8.33%)	1.00

A *p*-value< 0.05 indicates statistical significance (bold and italic)

Data presented as mean ± standard deviation or number and percentage

Abbreviations: *LVEF* Left ventricular ejection fraction, *LVEDV* Left ventricular end-diastolic volume, *LVESV* Left ventricular end-systolic volume, *LVMI* Left ventricular mass index, *LA area 2ch* Left atrial area from 2-chamber view, *LA length 2ch* Left atrial length from 2-chamber view, *LA area 4ch* Left atrial area from 4-chamber view, *LA length 4ch* Left atrial length from 4-chamber view, *LAVI* Left atrial volume index, *EPFR* Early peak filling rate, *LPFR* Late peak filling rate, *E velocity* Early diastolic filling velocity, *A velocity* Late atrial systolic filling velocity, *E/A* E/A ratio

The prevalence of LVDD was 20.7% (95% CI: 13.7–29.2%). Abnormal relaxation, pseudonormalization, and restrictive filling pattern was found in 3 (13%), 7 (29%), and 14 (58%) patients, respectively. Cardiac T2* was abnormal in 8.3% (95% CI: 1.0–27.0%) of patients in the LVDD group, and in 7.8% (95% CI: 3.6–14.2%) of all patients enrolled in this study.

Univariate and multivariate analysis was performed to assess for factors significantly associated with LVDD (Table 3). Age, body surface area (BSA), type of thalassemia (homozygous beta thalassemia), splenectomy, use of deferoxamine, heart rate (HR), diastolic blood pressure (DBP), and liver iron concentration were found to be univariately associated with LVDD. Factors found to be associated with LVDD in multivariate analysis were age (odds ratio [OR]: 1.07, 95% confidence interval [CI]: 1.02–1.12; *p* = 0.004), BSA (OR: 0.01, 95% CI: 0.0001–0.24; *p* = 0.005), homozygous beta-thalassemia (OR: 6.26, 95% CI: 1.38–28.39; *p* = 0.017), splenectomy (OR: 4.53, 95% CI: 1.29–15.93; *p* = 0.018), HR (OR: 0.92, 95% CI: 0.87–0.98; *p* = 0.008), and DBP (OR: 0.94, 95% CI: 0.89–0.99; *p* = 0.044).

**Table 3:** Univariate and multivariate analysis for factors associated with LV diastolic dysfunction

Factors	Univariate	*p*-value	Multivariate	*p*-value
	Crude OR (95% CI)		Adjusted OR (95% CI)	
Age	1.03 (1.00–1.06)	0.050	1.07 (1.02–1.12)	***0.004***
Body surface area	0.15 (0.02–1.52)	0.108	0.01 (0.0001–0.24)	***0.005***
Splenectomy	2.90 (1.11–7.68)	***0.030***	4.53 (1.29–15.93)	***0.018***
Homozygous beta-thalassemia	5.00 (1.55–16.11)	***0.007***	6.26 (1.38–28.39)	***0.017***
Deferoxamine	0.45 (0.15–1.31)	0.143	–	–
Beta blocker	3.14 (0.65–15.12)	0.153	–	–
Heart rate	0.93 (0.89–0.98)	***0.003***	0.92 (0.87–0.98)	***0.008***
Liver iron concentration	1.03 (0.99–1.07)	0.173	–	–
Diastolic blood pressure	0.95 (0.90–0.99)	***0.017***	0.94 (0.89–0.99)	***0.044***

A *p*-value< 0.05 indicates statistical significance (bold and italic)

Abbreviations: *LV* Left ventricular, *OR* Odds ratio, *CI* Confidence interval

Intraobserver and interobserver reliability was demonstrated by ICC, and the parameters were found to be well correlated (Table 4). All parameters were in good correlation.

**Table 4:** Intraclass correlation coefficient (*n* = 10)

Variable	Time 1 Mean ± SD	Time 2 Mean ± SD	Person 2 Mean ± SD	Intraobserver	*p*-value	Interobserver	*p*-value
				Time 1 vs. Time 2		Person 1 vs. Person 2	
EPFR (ml/s)	412.4 ± 105.4	433.6 ± 102.3	448.7 ± 104.9	0.99 (0.97–0.99)	***< 0.001***	0.97 (0.87–0.99)	***< 0.001***
LPFR (ml/s)	224.0 ± 73.8	224.9 ± 80.2	239.3 ± 81.7	0.99 (0.98–0.99)	***< 0.001***	0.97 (0.87–0.99)	***< 0.001***
DT (ms)	159.2 ± 25.8	160.4 ± 27.5	161.4 ± 26.6	0.97 (0.86–0.99)	***< 0.001***	0.94 (0.77–0.99)	***< 0.001***
E velocity (cm/s)	67.6 ± 12.4	67.6 ± 12.4	67.6 ± 12.4	1 (1–1)	NA	1 (1–1)	NA
A velocity (cm/s)	44.7 ± 12.5	44.7 ± 12.5	44.7 ± 12.5	1 (1–1)	NA	1 (1–1)	NA
E/A	1.6 ± 0.6	1.6 ± 0.6	1.6 ± 0.6	1 (1–1)	NA	1 (1–1)	NA
LA area 2ch (mm^2^)	18.2 ± 3.6	18.7 ± 3.9	18.5 ± 3.6	0.98 (0.93–0.99)	***< 0.001***	0.97 (0.89–0.99)	***< 0.001***
LA length 2ch (mm)	45.0 ± 5.4	44.9 ± 4.9	45.0 ± 5.6	0.95 (0.80–0.99)	***< 0.001***	0.86 (0.55–0.96)	***< 0.001***
LA area 4ch (mm^2^)	21.6 ± 5.5	22.1 ± 5.1	22.4 ± 4.8	0.98 (0.92–0.99)	***< 0.001***	0.98 (0.92–0.99)	***< 0.001***
LA length 2ch (mm)	55.1 ± 8.1	56.7 ± 8.0	51.2 ± 6.6	0.93 (0.73–0.98)	***< 0.001***	0.88 (0.60–0.97)	***< 0.001***
LA vol (ml)	75.3 ± 25.8	79.2 ± 79.4	79.4 ± 23.5	0.98 (0.91–0.99)	***< 0.001***	0.97 (0.90–0.99)	***< 0.001***
LAVI (ml/m^2^)	50.0 ± 15.0	52.5 ± 15.5	52.8 ± 13.3	0.97 (0.89–0.99)	***< 0.001***	0.99 (0.87–0.99)	***< 0.001***

A *p*-value< 0.05 indicates statistical significance (bold and italic)

Intraclass correlation coefficient (ICC) and 95% confidence interval

Abbreviations: *EPFR* Early peak filling rate, *LPFR* Late peak filling rate, *DT* Deceleration time, *E velocity* Early diastolic filling velocity, *A velocity* Late atrial systolic filling velocity, *E/A* E/A ratio, *LA area 2ch* Left atrial area from 2-chamber view, *LA length 2ch* Left atrial length from 2-chamber view, *LA area 4ch* Left atrial area from 4-chamber view, *LA length 4ch* Left atrial length from 4-chamber view, *LA vol* Left atrial volume, *LAVI* Left atrial volume index, *NA* Not available

We used CMR, not TTE, to determine LVDD in the present study. Therefore, we did not have data on TTE in every case. Additional analysis was performed to correlate CMR finding on diastolic dysfunction with TTE data among those who had available TTE results within 6 months prior to CMR examination. We demonstrated a good agreement of echocardiogram and CMR for the detection and correctly classified the grading of diastolic dysfunction in 17 patients (89.5%). Restrictive filling which is the most severe form of diastolic dysfunction was correctly detected in 100%.

## Discussion

The prevalence of LVDD in thalassemia major patients with normal LV systolic function in this study was 21.4%. Factors associated with LV diastolic dysfunction in multivariate analysis were age, BSA, DBP, HR, homozygous beta thalassemia, and splenectomy.

Previous studies showed that LVDD may relate to changes that occur in the early stage of cardiac iron overload, and that these changes may be detectable before systolic failure. The prevalence of LVDD in our study was more than that reported by Kremastinos, et al. [7], but less than that reported by Spirito, et al. [8] and Leonardi, et al. [9]. In the Kremastinos, et al. study [7], diastolic function by transthoracic echocardiograpgy (TTE) was compared between 88 beta-thalassemia major patients with normal LV systolic function and 46 normal control subjects. In that study, restrictive LV filling abnormalities were found in 7.9% (7/88) of patients (defined as increased E/A ratio, decreased DT, decreased S/D ratio [systolic to diastolic forward flow from pulmonary vein Doppler], and increased atrial reverse flow velocity [from pulmonary vein Doppler] with abnormal LV inflow and pulmonary vein flow patterns. None of those patients had abnormal relaxation (E/A ratio < 1 and prolonged isovolumic relaxation time). The prevalence of restrictive filling pattern was less than that found in our study (12%; 14/116 patients). This might be due to differences in diastolic dysfunction grading criteria between studies. Kremastinos, et al. used only restrictive filling pattern, which is the most severe form of diastolic dysfunction. The range of E/A ratio and DT in that study between the restrictive and non-restrictive groups was 2.12–4.14 vs. 1.06–2.14 and 105–125 ms vs. 120–190 ms, respectively. When these values (E/A ratio ≥ 2.12 and DT ≤125 ms) were used for diagnosis of restrictive filling pattern in our study, we found a prevalence was 5% (6/116 patients), which is close to that reported by Kremastinos, et al.

Spirito, et al. [8] reported the result of LV diastolic function assessed by TTE among 32 patients with thalassemia major with normal LV systolic function compared to 32 age and gender-matched normal subjects. In the patient group, there was significantly increased E wave, E/A ratio, and EF slope [rate of deceleration of flow velocity from the early diastolic peak (E)], and decreased DT, each of which reflecting a restrictive filling pattern [31–33]. That group used rather loose criteria, since any patient who had values of any one of these parameters beyond the 95% confidence limits of the control subjects were defined as abnormal diastolic function, which was demonstrated in 50% (16/32) of patients. As such, the high prevalence of LVDD in the Spirito, et al. study may be explained by differences in the diagnostic criteria between our study and theirs.

The relationship between CMR estimation of myocardial iron and LV systolic and diastolic function was studied in 24 transfusion-dependent thalassemia (TDT) patients with 47 paired TTE and CMR by Leonardi, et al. [9]. In that study, LVDD was defined, as follows: restrictive if E/A > 1.5 and DT < 140, impaired relaxation if E/A ≤ 0.75, and pseudonormalization if 0.75 < E/A < 1.5 and DT > 140 and E/E’ > 10. LV systolic function was evaluated by TTE and CMR. LV systolic function was normal in 32 cases by TTE and in 33 cases by CMR, mild dysfunction (LVEF: 41–55%) in 9 cases by TTE and in 7 cases by CMR, moderate dysfunction (LVEF: 31–40%) in 4 cases by TTE and in 6 cases by CMR, and severe dysfunction (LVEF: ≤30%) in 2 cases by TTE and in 1 case by CMR. Myocardial T2* was abnormal (< 20 ms) in 54% (13/24) of patients, and they found restrictive filling pattern (E/A ≥ 1.5 and DT < 140) in all study patients. We used the same criteria in our study and found a prevalence of restrictive filling pattern of 32% (37/116 patients), which is far lower than that reported by Leonardi, et al. This difference between studies may be explained by differences in baseline characteristics between the two study populations. Study population in Leonardi’s study were more severe disease as compared to patients enrolled in our study since they included patients with LV systolic dysfunction, a higher percentage of iron overload cardiomyopathy as shown by an abnormal T2*. In our study, all of the included patients had normal LV systolic function, and cardiac T2* was abnormal in 7.76% (95% CI: 3.61–14.22%).

We proposed a CMR criteria for the detection of LVDD modified from the ASE 2009 criteria [28], which focused on parameters on mitral valve filling, and LAVI as suggested by the ASE 2016 criteria [29]. We believed that many thalassemia patients were serially referred to CMR for the assessment of iron overload without request for echocardiogram. It would be better if we can assess cardiac iron overload, left ventricular systolic and diastolic function in the same setting with CMR.. E/A ratio, peak E velocity, deceleration time to grade LVDD in both ASE 2009 and current 2016 ASE guidelines. However, in 2016 ASE guideline, additional echocardiographic criteria were introduced (E/e’, peak TR velocity and LAV index) and some parameters are not available by CMR.

Previous data on prevalence of LVDD in patients with normal left ventricular systolic function showed that if we used the stringent criteria (only restrictive filling as reported by Kremastinos, et al), the prevalence would be 7.9% but is we used the loose criteria (any one of E, E/a, EF slope, or deceleration time being abnormal as reported by Spirito, et al), the prevalence would be 50%. The prevalence in our report should be more reasonable because our proposed CMR criteria are not too stringent and not too loose.

Age, obesity, and hypertension are known to be associated with diastolic dysfunction [34, 35]; however, the factors associated with LVDD in thalassemia major patients are not well defined. The main factors that were associated with LVDD were homozygous beta-thalassemia and splenectomy. Patients with homozygous beta-thalassemia had the risk of LVDD of 50% whereas beta-thalassemia/Hb E had the risk of LVDD of 17% (*p* = 0.004). Patients with splenectomy had the risk of LVDD of 36% compared to the risk of 16% for those without splenectomy (*p* = 0.024). When we combined the effect of these 2 factors, patients with beta-thalassemia/Hb E and no splenectomy had the risk of LVDD of 12%. The risk increased to 30, 44 and 60% for beta-thalassemia/Hb E with splenectomy, homozygous beta-thalassemia without splenectomy and homozygous beta-thalassemia with splenectomy respectively. The effect of splenectomy and homozygous beta-thalassemia on the prevalence of LVDD was mainly in population without cardiac iron overload since our population were well treated with chelation therapy. No correlation was found between LV diastolic function and cardiac T2*. The prevalence of abnormal cardiac T2* defined as lower than 20 ms was only 8% which is too small to make conclusion for the relation of cardiac iron overload and LVDD and other factors. However, when we looked at data of 4 patients with severe cardiac iron overload (defined as cardiac T2* less than 10 ms), the prevalence of LVDD in this group was 50% which is much higher than 20% those with cardiac T2* less than 10 ms but the number is too small to achieve statistical significance. Patients with homozygous beta-thalassemia had an increased risk of cardiac iron overload compared to beta-thalassemia/Hb E (21% vs 6%, *p* = 0.041). Patients with splenectomy also had an increased trend of cardiac iron overload (11% vs 7%, p = NS). Splenectomy results in loss of function to remove hematologic debris and to suppress intravascular hemolysis, which leads to increased oxidation, procoagulative state, vasoconstricting substances, smooth muscle proliferation, and endothelial dysfunction [36]. As an alternative to the propose mechanism of iron overload cardiomyopathy only, the mechanism of LVDD in thalassemia major may be due to these pathology in splenectomy patients. Association between homozygous beta thalassemia and LVDD could be explained by splenectomy, which was performed in patients with homozygous beta thalassemia more than in patients with Hb E/beta-thalassemia. Both homozygous beta-thalassemia and splenectomy reflect a more severe disease that could lead to LVDD despite no evidence of cardiac iron overload detected by cardiac T2*. Therefore, even in patients without detectable cardiac iron overload, LVDD should be looked for in order to detect early cardiac involvement. Early treatment may be possible.

Decrease in BSA associated with more LVDD. BSA may reflect a patient’s nutritional status, since vitamin B, vitamin D, and carnitine deficiency were reported to be associated with impaired cardiac function [30]. HR and DBP were also associated with LVDD, but the mechanism could not be defined. The following theory might explain the association of low DBP and LVDD. As arterial stiffness increases, the pulse wave velocity along the aorta increases, so that the reflected pulse wave arrives earlier at the ascending aorta and augments the late-systolic ascending aortic pressure waveform and decrease diastolic pressure [37]. Furthermore, late systolic arterial load to the left ventricle, which results in deteriorated left ventricular relaxation [38]. Low DBP has been reported in patients with beta-thalassemia major compared to controls [39]. It has also been reported in other hemolytic disease such as sickle cell anemia [40]. They also measured aortic stiffness parameters and found an increased aortic stiffness in patients with low DBP just like what we just mentioned. An abnormality in autonomic balance as measured by heart rate variability indicated an increase in vagal tone was also demonstrated which may be related to a decrease heart rate in patients with LVDD [39].

This study has some limitations. First, we cannot extrapolate the results since we enrolled only thalassemia patients who were requested for cardiac T2*. Second, we did not have echocardiographic data to correlate with CMR findings in every patient. We have echocardiographic data in only 19 patients. Although echocardiogram and CMR was not performed on the same day, we could demonstrate a good correlation with CMR for the detection and grading of diastolic dysfunction in 89.5%. E and A values had moderate positive relationship between TTE and CMR methods. Both E and A values from TTE were higher than those measured by CMR, which is similar to previous study [24]. A strong positive relationship was observed between E/A ratio and LAVI. Third and last, mitral valve flow measurement by CMR may yield lower values for the E and A parameters than those reported in echocardiographic studies, because of the method of velocity measurement might not be at the tip of the mitral valve.

Further prospective studies of the left ventricular diastolic function by CMR and the cardiac iron overload in various stages of the disease since diagnosis and follow up during treatment may yield more understanding of the disease physiology. Future research for the effect of early detection and treatment on the progression of disease and prognosis is encouraged.

## Conclusion

LVDD already exists from the early stages of the disease before the abnormal heart T2 * is detected. Homozygous beta-thalassemia and splenectomy were strong predictors of LVDD. These data may increase awareness of the disease, especially in the high risk groups.

## Data Availability

The dataset that was used to support the conclusion of this study is included within the manuscript. Any additional data will be made available upon the request to Rungroj Krittayaphong at rungroj.kri@mahidol.ac.th.

## Abbreviations

CI: Confidence interval
CMR: Cardiac magnetic resonance
DBP: Diastolic blood pressure
DT: Deceleration time
EPFR: Early peak filling rate
HR: Heart rate
ICC: Intraclass correlation coefficient
LA: Left atrial
LAVI: Left atrial volume index
LPFR: Late peak filling rate
LV: Left ventricular
LVDD: Left ventricular diastolic dysfunction
OR: Odds ratio
